# Supplementary material for: Illusory finger stretching and somatosensory responses in participants with chronic hand-based pain
Source: PLoS One. 2025 Feb 4;20(2):e0317693. doi: 10.1371/journal.pone.0317693 (PMC11793786; doi:10.1371/journal.pone.0317693)
Supplement: S1 Fig — (PDF) [file pone.0317693.s001.pdf]

Previous literature states that the ideal vibration frequency to use to elicit somatosensory steady state evoked potentials (SSSEPs) ranges from 26-27Hz [1,2,3,4]. Due to resizing illusions often manipulating the index finger, and previous studies using the index finger supporting around 26Hz as an optimal frequency [1,2,3], it was hypothesised that 26Hz would elicit a dependable SSSEP. Therefore, we ran a pilot study to check that our setup and equipment can reliably elicit and record a SSSEP at 26Hz, using the resizing illusion and EEG.

Pilot data was collected for 3 healthy participants. Participants underwent the same experimental protocol as mentioned in the “Experimental Procedure” section, minus the subjective illusory experience and pain rating scales. No additional filtering or denoising steps were applied to the EEG data, in line with Figueira et al.’s [5] report that only a Fourier transform is typically needed for this type of EEG data. A Fourier transform was calculated for each waveform at each electrode for all conditions, and then averaged across repetition to obtain individual results. These were then averaged across all 3 participants to give the result seen in Figure 3.

As can be seen, there is a clear SSSEP response at 26Hz, which is strongest around electrodes F1 and FC1. Previous research using vibrotactile 21Hz stimulation have also found the scalp topography of the activation to be most pronounced over mid-frontal distributions [6,7], in line with the scalp topography seen here. Given these finding of a distinct 26Hz signal and mid-frontal scalp location, it appears appropriate for 26Hz to be used as the vibration frequency in the proposed study.

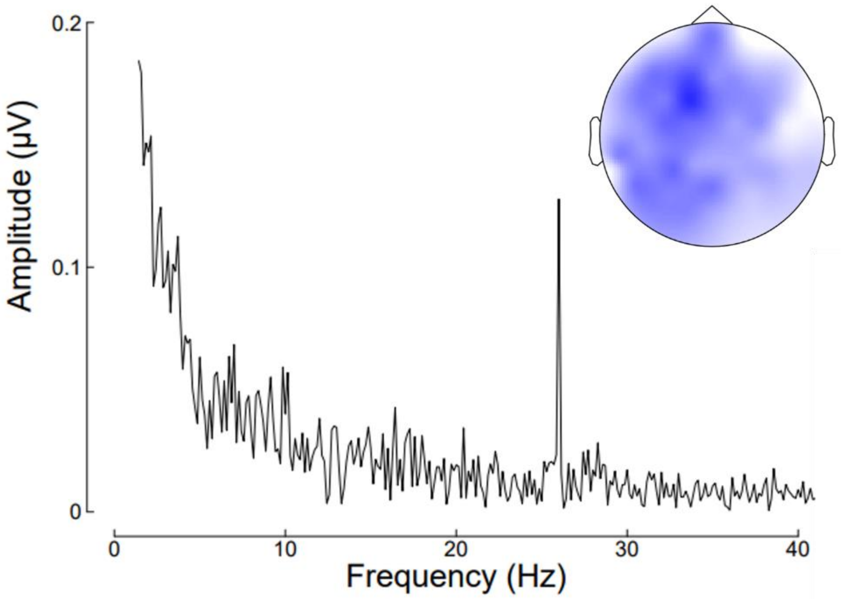

***S1 Fig*. Averaged Pilot Data showing peak frequency at 26Hz, centred between electrodes F1 and FC1.** The spectrum is derived from electrode FC1. Saturation bar represents signal to noise ratio (SNR). SNR is a measure of signal quality and describes the ratio of signal power (at 26Hz) to noise power (averaged across 10 adjacent frequency bins). SNR was used for the pilot figure because with a small sample (3 participants) we did not want a noisy electrode to influence the electrodes chosen as electrodes of interest.

1. Muller GR, Neuper Ch, Pfurtscheller G. „Resonance-like“ Frequencies of Sensorimotor Areas Evoked by Repetitive Tactile Stimulation—Resonanzeffekte in sensomotorischen Arealen, evoziert durch rhythmische taktile Stimulation. Biomed Tech (Berl). 2001;46(7–8):186–90. https://doi.org/10.1515/bmte.2001.46.7-8.186
2. Breitwieser C, Pokorny C, Müller-Putz GR. A hybrid three-class brain–computer interface system utilizing SSSEPs and transient ERPs. J Neural Eng. 2016;13(6):066015. https://doi.org/10.1088/1741-2560/13/6/066015
3. Pokorny C, Breitwieser C, Müller-Putz GR. The role of transient target stimuli in a steady-state somatosensory evoked potential-based brain–computer interface setup. Front Neurosci. 2016;10. https://doi.org/10.3389/fnins.2016.00152
4. Snyder AZ. Steady-state vibration evoked potentials: Description of technique and characterization of responses. Electroencephalogr Clin Neurophysiol. 1992;84(3):257–68. https://doi.org/10.1016/0168-5597(92)90007-X
5. Figueira JSB, Kutlu E, Scott LS, Keil A. The FreqTag toolbox: A principled approach to analyzing electrophysiological time series in frequency tagging paradigms. Dev Cogn Neurosci. 2022;54:101066. https://doi.org/10.1016/j.dcn.2022.101066
6. Porcu E, Keitel C, Müller MM. Visual, auditory and tactile stimuli compete for early sensory processing capacities within but not between senses. Neuroimage. 2014;97:224–35. https://doi.org/10.1016/j.neuroimage.2014.04.024
7. Timora JR, Budd TW. Steady-state EEG and psychophysical measures of multisensory integration to cross-modally synchronous and asynchronous acoustic and vibrotactile amplitude modulation rate. Multisensory Res. 2018;31(5):391–418. https://doi.org/10.1163/22134808-00002549
