# Supplementary material for: Illusory finger stretching and somatosensory responses in participants with chronic hand-based pain
Source: PLoS One. 2025 Feb 4;20(2):e0317693. doi: 10.1371/journal.pone.0317693 (PMC11793786; doi:10.1371/journal.pone.0317693)
Supplement: S2 Fig — (PDF) [file pone.0317693.s002.pdf]

Pilot data was also collected using the vibrotactile stimulator at 26Hz to make sure that the illusory experience is not removed due to the addition of this vibrotactile input. Pilot data was collected from 4 additional healthy participants, who underwent the same experimental protocol as mentioned in the "Experimental Procedure" section, simply without EEG caps fitted, and without pain scales used. Illusory experience was calculated using the average of both illusion scores for each participant, and then averaging over participants to give the results seen in Figure 4. As can be seen, there is a greater subjective experience of the resizing illusion, indexed by participant's illusion score, in both experimental conditions (UV average = 64.25; MS average = 67.88) compared to both control conditions (NI average = 32.38; NIT average = 24.13). Scores below 50 are indicative of disagreement of experience of the illusion, whilst a score of 50 is a neutral option regarding the illusion experience, and scores above 50 are indicative of agreement of experiencing the illusion. This therefore shows that the addition of the vibrotactile stimulation does not remove the experience of the resizing illusion and can therefore be used in the proposed study to elicit SSEPs without affecting the subjective illusory experience of the resizing illusion.

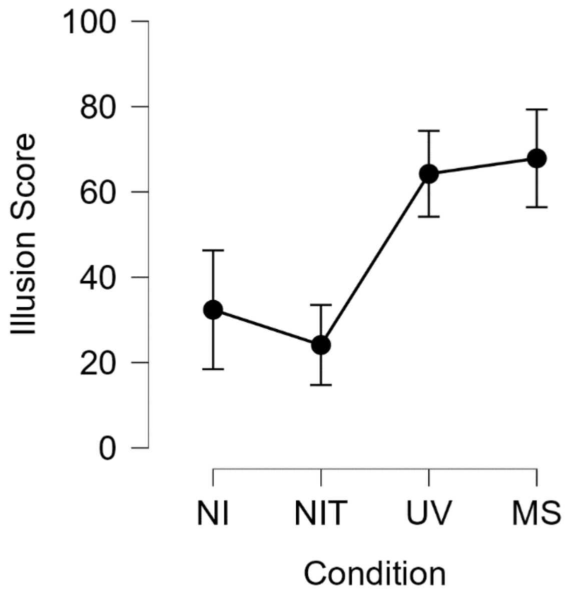

S2 Fig. Averaged Illusion score for each condition. Error bars represent standard errors.
