# Supplementary material for: Illusory finger stretching and somatosensory responses in participants with chronic hand-based pain
Source: PLoS One. 2025 Feb 4;20(2):e0317693. doi: 10.1371/journal.pone.0317693 (PMC11793786; doi:10.1371/journal.pone.0317693)
Supplement: S3 Fig — (PDF) [file pone.0317693.s003.pdf]

Exploratory analyses of the subjective disownership data using a Friedman test found a significant overall effect of condition with a small to moderate effect size (χ 2 (3) = 13.12, p = 0.004, Kendall's W = 0.21) and post hoc Wilcoxon tests with Holm corrections found significantly greater combined disownership score in the UV condition (Median = 42, SD = 42.32) compared to the NI (Median = 0, SD = 19.63, *z* = 16, *p.adj* = 0.039, *r* = -39.78), NIT (Median = 0, SD = 15.24, *z* = 12, *p.adj* = 0.025, *r* = -48.25), and MS conditions, (Median = 1.5, SD = 22.34, *z* = 22, *p.adj* = 0.042, *r* = -40).

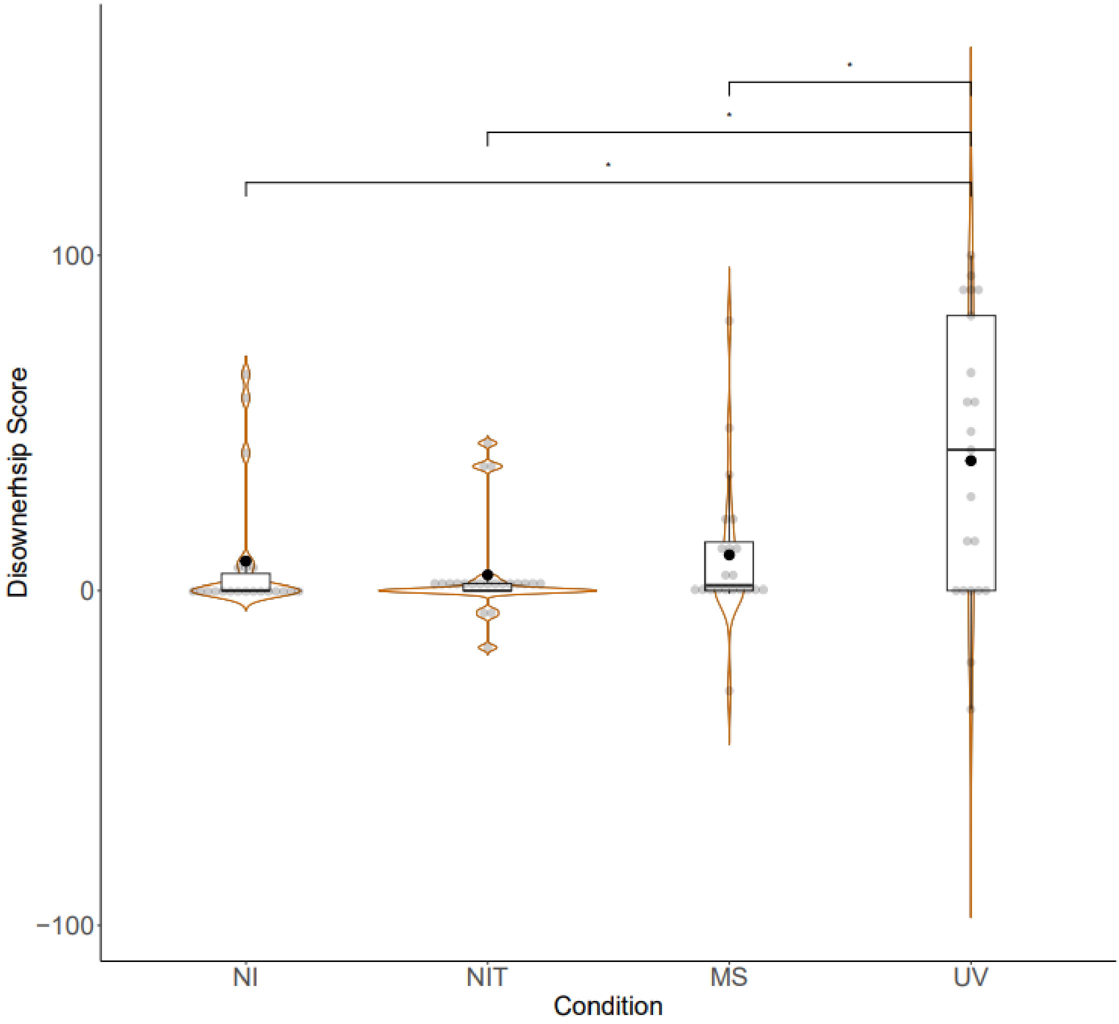

S3 Fig. Combined Disownership Score Index Across Conditions (NI: Non-Illusion; NIT: Non-Illusion Tactile; MS: Multisensory; UV: Unimodal Visual). Scores below 50 indicate disagreement with experience of disownership statements, whilst scores above 50 indicate agreement. A continuous visual analogue scale was used in data collection, with agreement and disagreement statements located at each end of the scale. Box plots show means, medians and inter-quartile ranges of data. Medians are indicated with a horizontal line whilst means are indicated by a black dot. Data points are shown in grey jitter binned along the y-axis, grouped by condition.
