# Supplementary material for: Illusory finger stretching and somatosensory responses in participants with chronic hand-based pain
Source: PLoS One. 2025 Feb 4;20(2):e0317693. doi: 10.1371/journal.pone.0317693 (PMC11793786; doi:10.1371/journal.pone.0317693)
Supplement: S4 Fig — (PDF) [file pone.0317693.s004.pdf]

Exploratory analyses of the subjective control data using a Friedman test found a significant overall effect of condition with a small effect size (χ 2 (3) = 11.61, p = 0.009, Kendall's W = 0.18). Post hoc Wilcoxon tests with Holm corrections for multiple comparisons found a significant difference between NI and UV control scores (*z* = 1, *p.adj* = 0.031, *r* = -16) however found no significant differences between control scores across any other condition: NI (Median = 0, SD = 21.8), NIT (Median = 0, SD = 9.2), MS (Median = 0, SD = 17.28), UV (Median = 6, SD = 24.02).

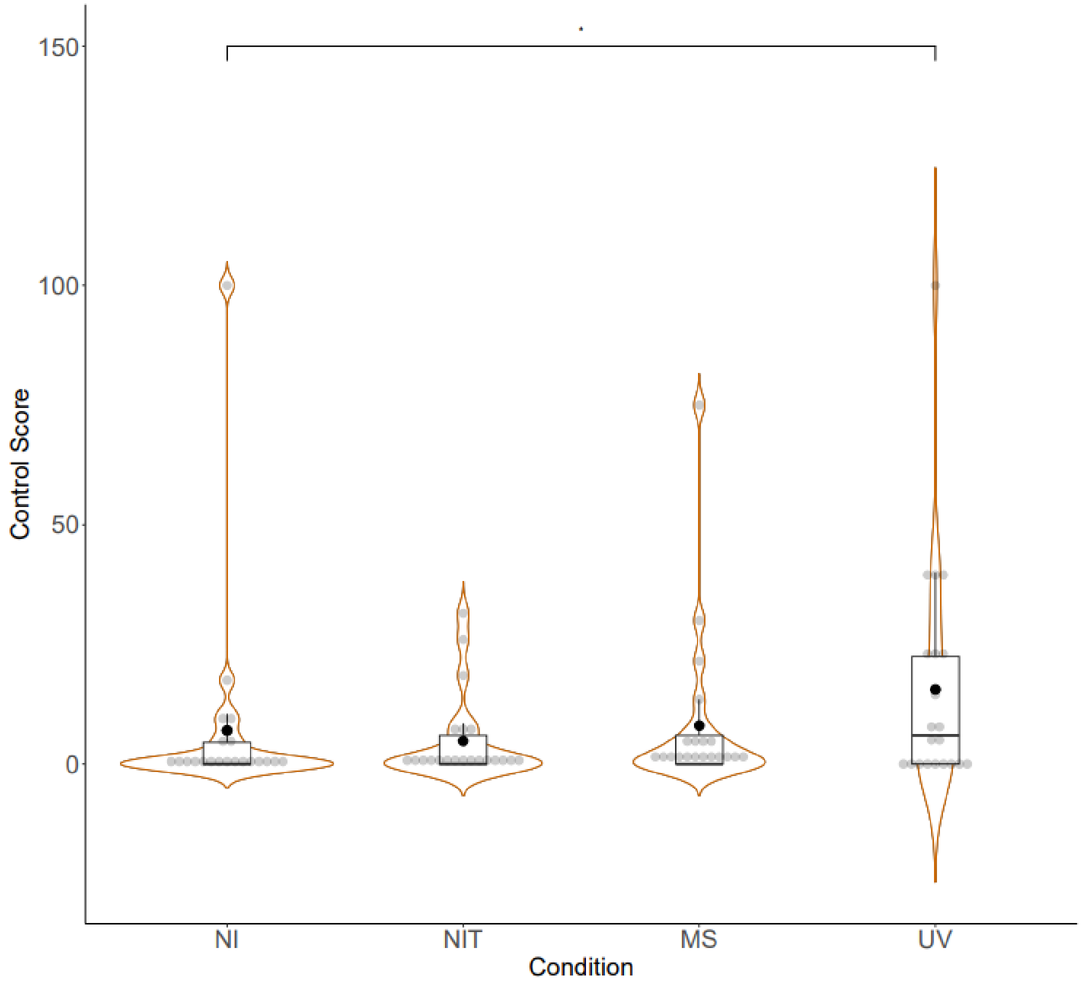

S4 Fig. Combined Control Scores Across Conditions (NI: Non-Illusion; NIT: Non-Illusion Tactile; MS: Multisensory; UV: Unimodal Visual). Scores below 50 indicate disagreement with experience of control statements, whilst scores above 50 indicate agreement. A continuous visual analogue scale was used in data collection, with agreement and disagreement statements located at each end of the scale. Box plots show means, medians and inter-quartile ranges of data. Medians are indicated with a horizontal line whilst means are indicated by a black dot. Data points are shown in grey jitter binned along the y-axis, grouped by condition.
