# Supplementary material for: Illusory finger stretching and somatosensory responses in participants with chronic hand-based pain
Source: PLoS One. 2025 Feb 4;20(2):e0317693. doi: 10.1371/journal.pone.0317693 (PMC11793786; doi:10.1371/journal.pone.0317693)
Supplement: S5 Fig — (PDF) [file pone.0317693.s005.pdf]

Spearman's correlations were run to identify any correlations between (1) participant's illusion score and their SSEP amplitude, (2) participant's pain percentage change and their SSEP amplitude, and (3) participant's subjective illusion scores and their pain percentage change, finding no significant correlations across any analyses.

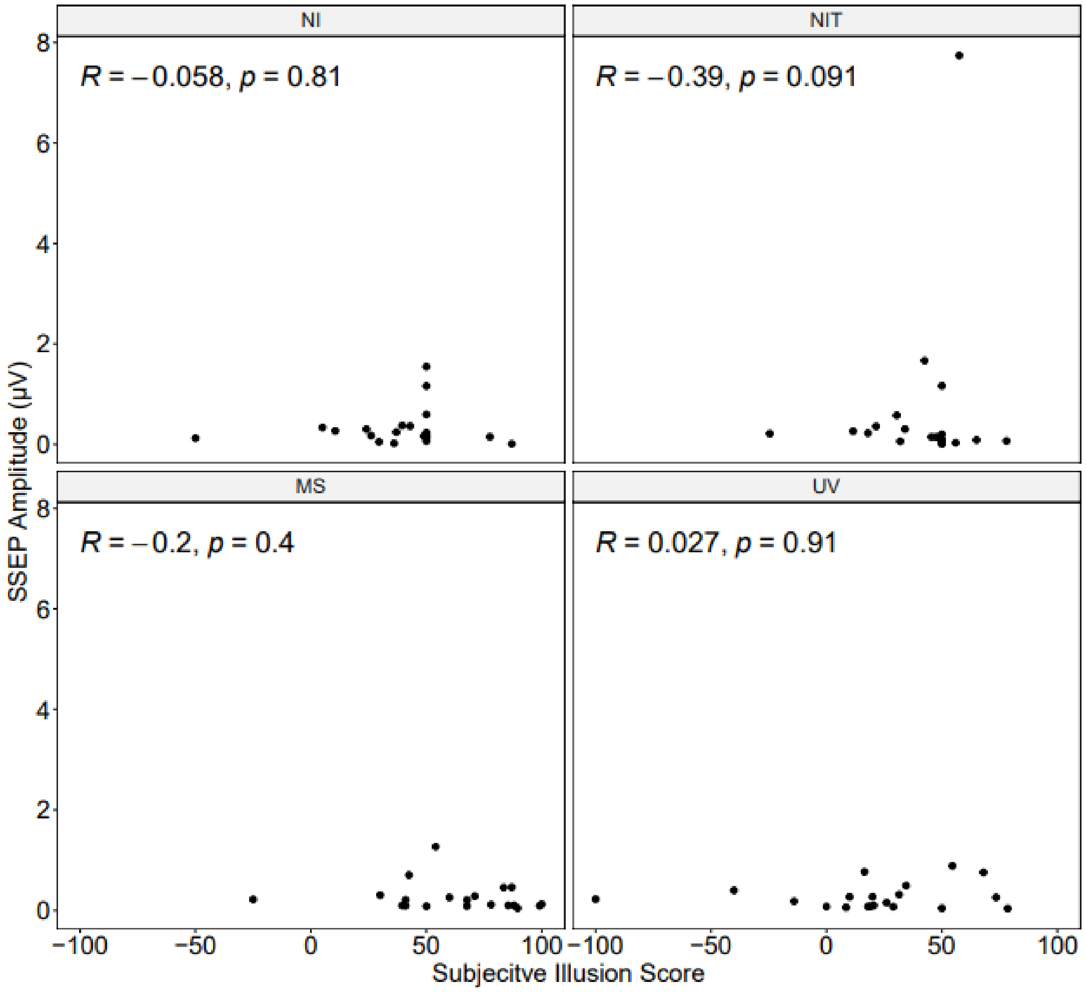

S5 Fig. Correlation Between Amplitude and Subjective Illusory Score for Each Condition.
