# Supplementary figures and images for: Illusory finger stretching and somatosensory responses in participants with chronic hand-based pain
Source: PLoS One. 2025 Feb 4;20(2):e0317693. doi: 10.1371/journal.pone.0317693 (PMC11793786; doi:10.1371/journal.pone.0317693)

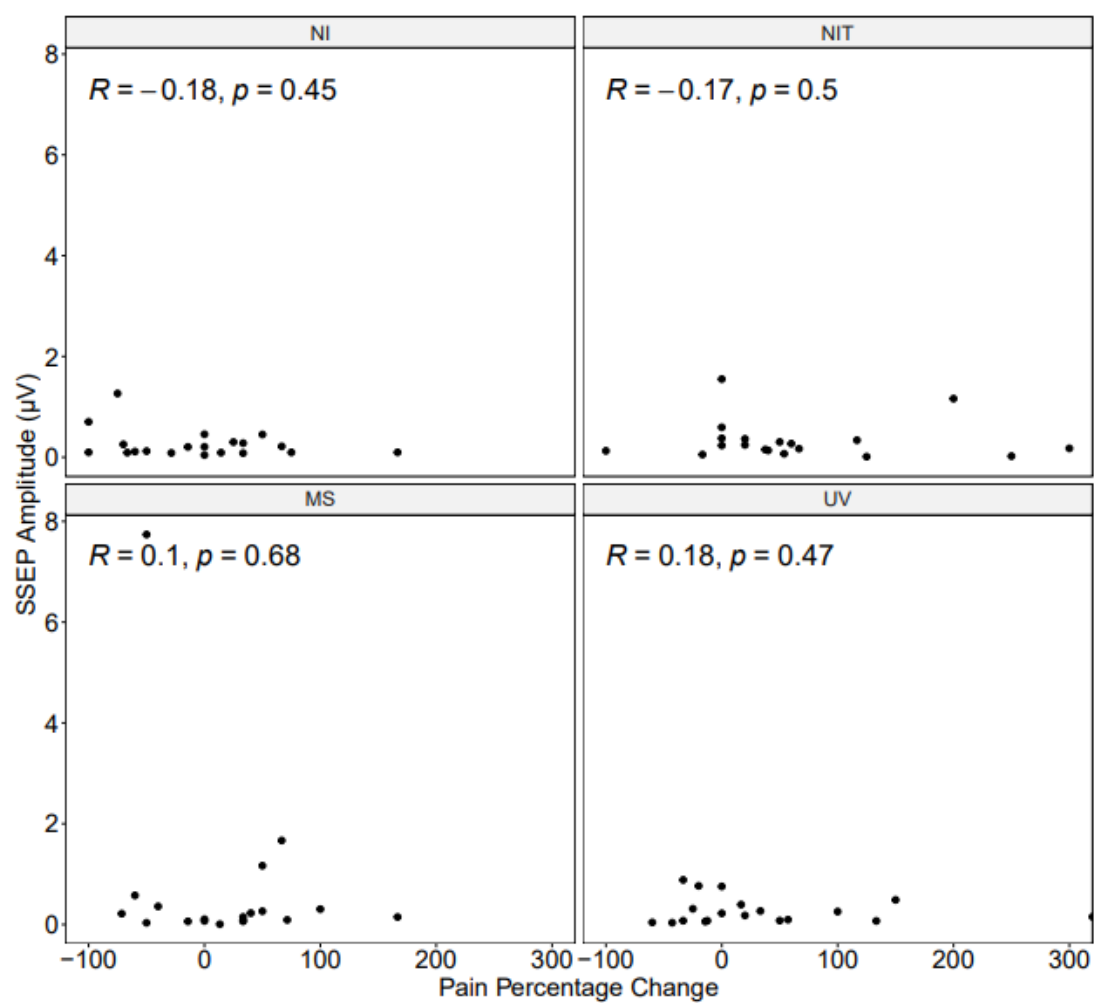

**S6 Fig. Correlation Between Amplitude and Pain Percentage Change for Each Condition.**

Supplement: S6 Fig — (PDF) [file pone.0317693.s006.pdf]
