# Supplementary material for: Illusory finger stretching and somatosensory responses in participants with chronic hand-based pain
Source: PLoS One. 2025 Feb 4;20(2):e0317693. doi: 10.1371/journal.pone.0317693 (PMC11793786; doi:10.1371/journal.pone.0317693)
Supplement: S7 Fig — (PDF) [file pone.0317693.s007.pdf]

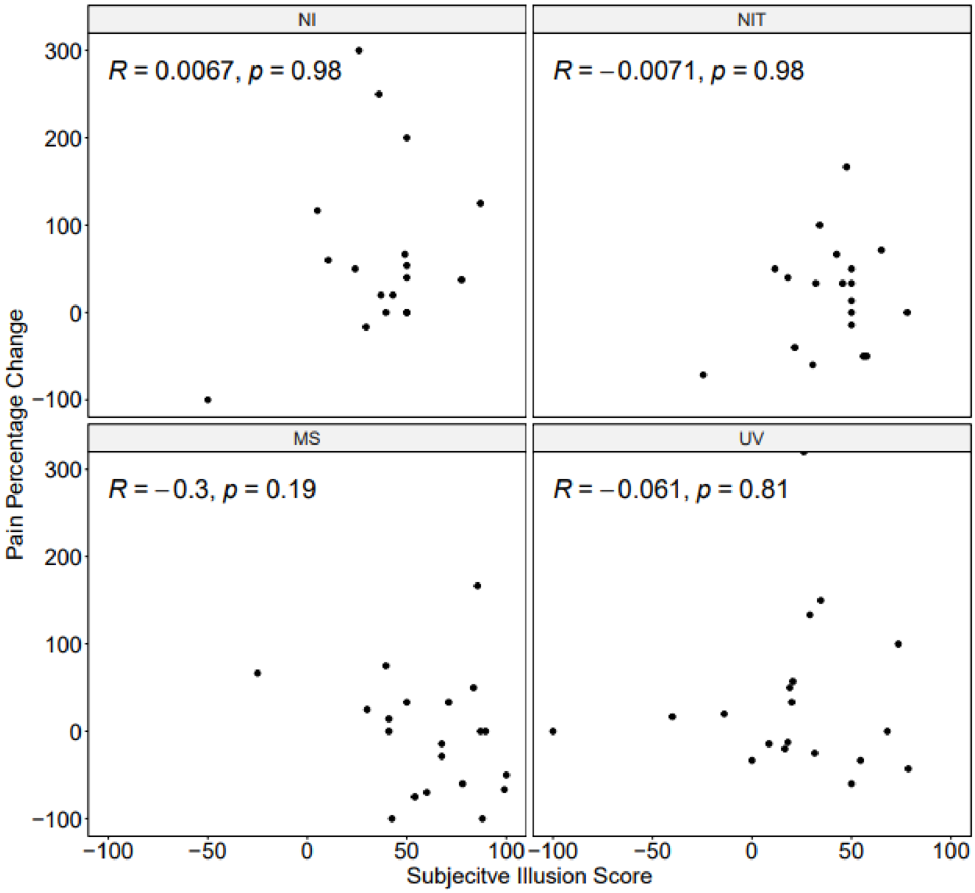

S7 Fig. Correlation Between Pain Percentage Change and Subjective Illusory Score for Each Condition.
