# Supplementary material for: Illusory finger stretching and somatosensory responses in participants with chronic hand-based pain
Source: PLoS One. 2025 Feb 4;20(2):e0317693. doi: 10.1371/journal.pone.0317693 (PMC11793786; doi:10.1371/journal.pone.0317693)
Supplement: S8 Fig — (PDF) [file pone.0317693.s008.pdf]

Since this sample consisted of participants with both primary and secondary chronic pain conditions, data were analysed split by either primary or secondary pain condition and can be seen in the figures below. Chronic primary pain is plotted in magenta, chronic secondary pain is plotted in green, and participants with either no diagnosis or a mix of primary and secondary pain conditions are plotted in grey.

Regarding chronic primary pain conditions, Wilcoxon tests found no significant differences when comparing NI pre (Median = 5, SD = 1.41) and post (Median = 6, SD = 0.87) pain levels (z = 13, *p.adj* = 0.170, r = 1.25), nor when comparing NIT pre (Median = 5, SD = 1.48) and post (Median = 7, SD = 2.28) pain levels (z = 8, *p.adj* = 0.345, r = 2), MS pre (Median = 4, SD = 2.88) and post (Median = 5, SD = 2.61) levels (z = 6, *p.adj* = 0.855, r = 1.14) nor UV pre (Median = 6, SD = 0.84) and post (Median = 7, SD = 2.74) levels (z = 10, *p.adj* = 0.586, r = 1).

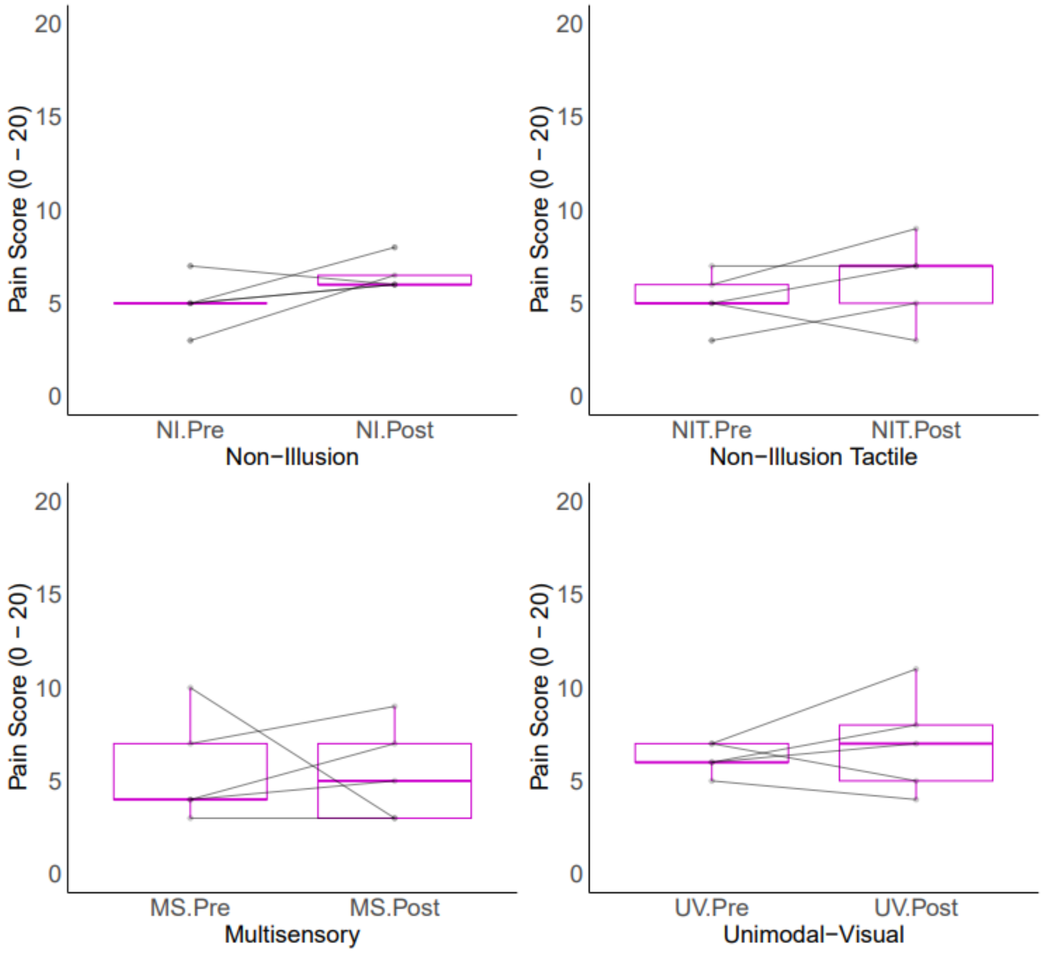

S8 Fig. Pre and Post Pain Scores Across Conditions for Participants with Chronic Primary Pain. Box plots show medians and inter-quartile ranges of data. Paired data points are shown in grey.
