# Supplementary material for: Illusory finger stretching and somatosensory responses in participants with chronic hand-based pain
Source: PLoS One. 2025 Feb 4;20(2):e0317693. doi: 10.1371/journal.pone.0317693 (PMC11793786; doi:10.1371/journal.pone.0317693)
Supplement: S9 Fig — (PDF) [file pone.0317693.s009.pdf]

No differences in pain levels were found for chronic secondary pain conditions when comparing NI pre (Median = 4.5, SD = 3.47) and post (Median = 6, SD = 3.7) pain levels (*z* = 13.5, *p.adj* = 0.595, *r* = 13.5), nor when comparing NIT pre (Median = 3.5, SD = 2.57) and post (Median = 2, SD = 3.43) pain levels (*z* = 15, *p.adj* = 0.932, *r* = 15), MS pre (Median = 4.5, SD = 3.06) and post (Median = 3.5, SD = 3.62) levels (*z* = 16, *p.adj* = 0.474, *r* = 16) nor UV pre (Median = 3.5, SD = 2.85) and post (Median = 3, SD = 2.37) levels (*z* = 8.5, *p.adj* = 0.386, *r* = 8.5).

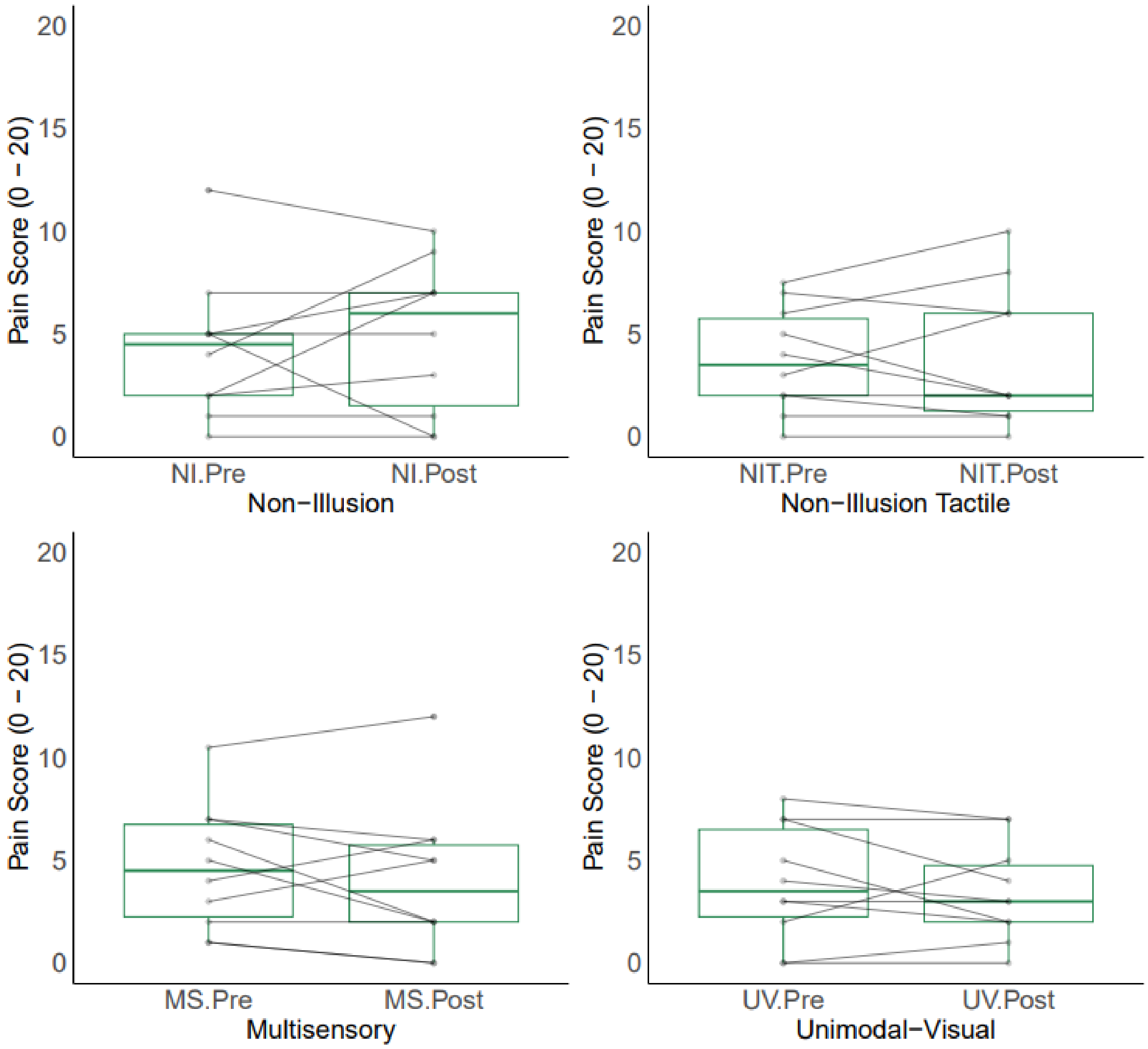

S9 Fig. Pre and Post Pain Scores Across Conditions for Participants with Chronic Secondary Pain. Box plots show medians and inter-quartile ranges of data. Paired data points are shown in grey.
