# Supplementary material for: Illusory finger stretching and somatosensory responses in participants with chronic hand-based pain
Source: PLoS One. 2025 Feb 4;20(2):e0317693. doi: 10.1371/journal.pone.0317693 (PMC11793786; doi:10.1371/journal.pone.0317693)
Supplement: S10 Fig — (PDF) [file pone.0317693.s010.pdf]

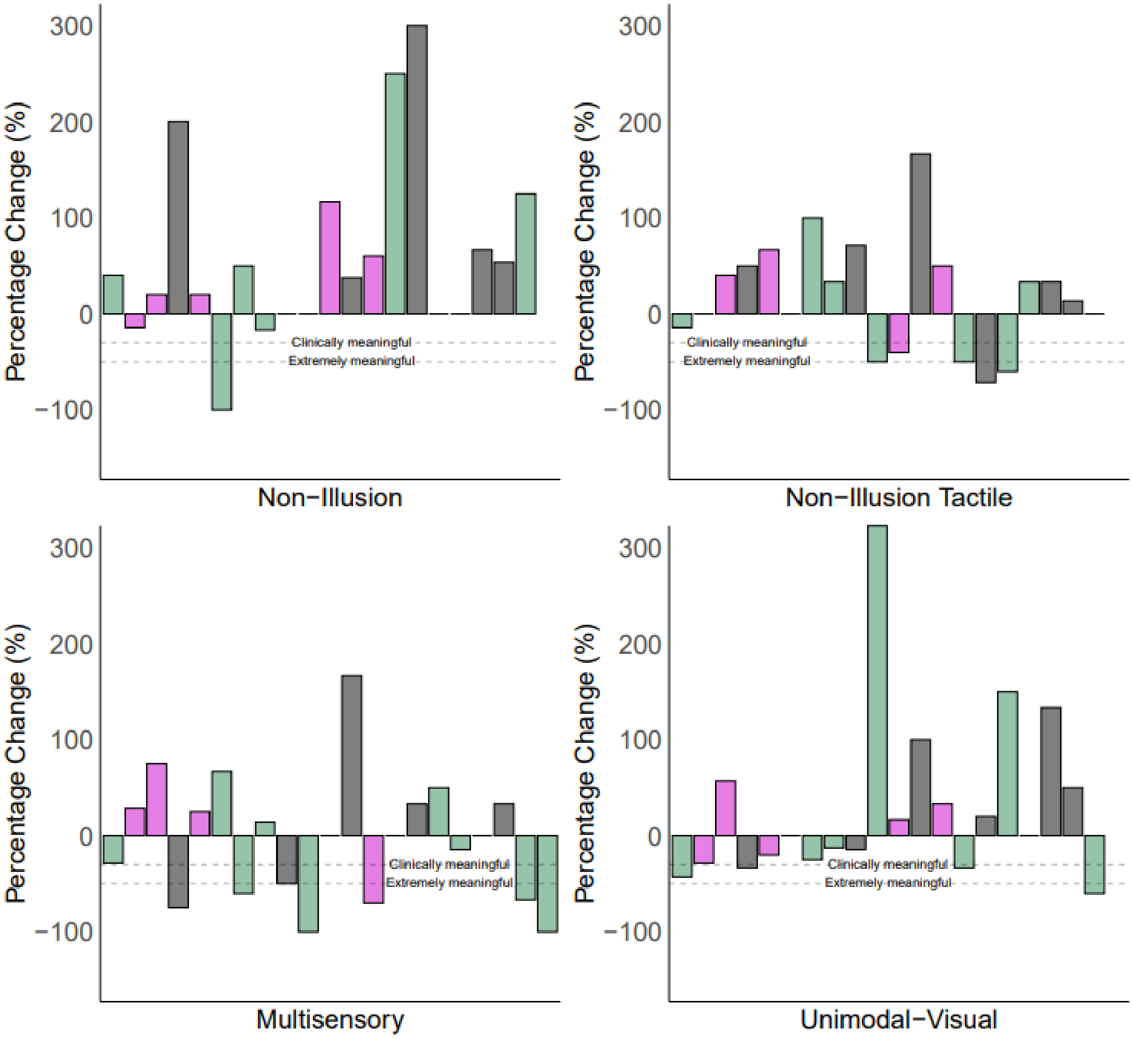

S10 Fig. Percentage change for pain scores across all conditions per participant. Dashed lines show 30% pain reduction (clinically meaningful) and 50% pain reduction (extremely meaningful). Bars showing in magenta show participants with chronic primary pain, green for those with chronic secondary pain, and grey for participants with either no diagnosis or a mix of primary and secondary pain conditions.
