# Supplementary material for: Illusory finger stretching and somatosensory responses in participants with chronic hand-based pain
Source: PLoS One. 2025 Feb 4;20(2):e0317693. doi: 10.1371/journal.pone.0317693 (PMC11793786; doi:10.1371/journal.pone.0317693)
Supplement: Attachment — Submitted filename: Response to Reviewers.docx [file pone.0317693.s011.docx]

Reply to Reviewers Comments

The authors would like to thank the reviewers and the editor for their time and comments on our manuscript, we have addressed each point in italics below the comment raised (in bold). New text is indicated with an underline.

**1. Please ensure that your manuscript meets PLOS ONE's style requirements, including those for file naming. The PLOS ONE style templates can be found at** 
[**https://journals.plos.org/plosone/s/file?id=wjVg/PLOSOne_formatting_sample_main_body.pdf**](https://journals.plos.org/plosone/s/file?id=wjVg/PLOSOne_formatting_sample_main_body.pdf) **and** 
[**https://journals.plos.org/plosone/s/file?id=ba62/PLOSOne_formatting_sample_title_authors_affiliations.pdf**](https://journals.plos.org/plosone/s/file?id=ba62/PLOSOne_formatting_sample_title_authors_affiliations.pdf)

*The manuscript, supporting information, and all associated files have been updated to reflect the style requirements shown in the above templates.*

**2. Thank you for stating the following in the Acknowledgments Section of your manuscript: 
The authors would like to thank the Pain Relief Foundation for funding this research, and B.P.A Quinn for production of the schematic used in Figure 1. We would also like to thank all the participants who gave their time to take part in this study.**

**We note that you have provided funding information that is not currently declared in your Funding Statement. However, funding information should not appear in the Acknowledgments section or other areas of your manuscript. We will only publish funding information present in the Funding Statement section of the online submission form. 
Please remove any funding-related text from the manuscript and let us know how you would like to update your Funding Statement. Currently, your Funding Statement reads as follows: 
 A Pain Relief Foundation (**[**https://painrelieffoundation.org.uk/**](https://painrelieffoundation.org.uk/)**) John Miles PhD Studentship grant funded the work for this manuscript. The grant was awarded to C.E.J.P. The funders did not play any role in study design, data collection or analysis, or the preparation of the manuscript.**

**Please include your amended statements within your cover letter; we will change the online submission form on your behalf.**

*The mention of funding has now been removed from the acknowledgements section. Regarding the funding statement, the John Miles PhD Studentship is the funding that was referred to in the acknowledgements section, therefore there is no need to update the funding statement as all information is correct.*

**3. Please include captions for your Supporting Information files at the end of your manuscript, and update any in-text citations to match accordingly. Please see our Supporting Information guidelines for more information:** [**http://journals.plos.org/plosone/s/supporting-information**](http://journals.plos.org/plosone/s/supporting-information)**.**

*Captions for all supporting information figures have now been included at the end of the manuscript (after the references section) and all in-text citations relating to these figures have been updated in the manuscript to reflect the guidelines mentioned above.*

**4. Please review your reference list to ensure that it is complete and correct. If you have cited papers that have been retracted, please include the rationale for doing so in the manuscript text, or remove these references and replace them with relevant current references. Any changes to the reference list should be mentioned in the rebuttal letter that accompanies your revised manuscript. If you need to cite a retracted article, indicate the article’s retracted status in the References list and also include a citation and full reference for the retraction notice.**

*The reference list has been reviewed and edited – The R core team reference was in the wrong place so has been moved, which now makes all subsequent references correctly aligned, the Hansford et al references [21] has been updated to reflect the published version of the manuscript rather than the preprint, and finally the references at the end of the list which referred to those used within the supporting information S1 file have been removed from the main manuscript document text and have been added to the S1.pdf file. To our knowledge there are no references included within the reference list which refer to a retracted manuscript.*

**Reviewer #1:**

**• There is conflicting information between the description of chronic pain diagnosis (primary, secondary, and non-diagnosed) in lines 154-155 and the inclusion criteria in lines 169-170, which state that no formal diagnosis was required. Was any type of diagnosis conducted before the study? If no formal diagnosis was performed and participants with non-diagnosed chronic pain were included based on self-reported joint pain lasting three or more months, this should be stated more explicitly to clarify the characteristics of the study population.**

*More detail has been added to the inclusion criteria section to say that diagnoses were collected before participants took part, and that these were then used to categorise participants into groups for the exploratory analyses as can be seen below:*

*“*All participants were asked whilst giving consent to take part if they had any chronic pain condition diagnoses. Diagnoses were then categorised into either chronic primary pain conditions, chronic secondary pain conditions, those with a mixture of primary and secondary conditions, and those without a diagnosis for the purpose of exploratory chronic pain condition analyses.”* **• If no formal diagnosis of chronic pain was required for participation in the study, this would imply a significant derivation from the inclusion criteria stated in the pre-registered document at** [**https://osf.io/9anjc**](https://osf.io/9anjc)**. Including a section indicating the possible derivations from the pre-registered document, including the decision not to acquire further datasets after excluding a participant, would be desirable to reinforce the power and relevance of the results presented in this manuscript.**

*A new section has been added to note deviations from the preregistration:*

## *“****2.1 Preregistratrion***

*This study was preregistered at the following OSF page: https://osf.io/9anjc . Deviations from the preregistration are as follows:*

*- To prevent bias towards participants with a diagnosis of a chronic pain condition, participants without diagnoses were recruited as long as they self reported to be experiencing ongoing or reoccurring pain for more than 3 months.*

*- Due to scarcity of participants eligible to take part in this project within the timeframe, additional participants were not recruited to replace any lost due to incomplete or noisy data.”*

**• The reviewers understand that a subset of participants was excluded because they had been previously diagnosed with Complex Regional Pain Syndrome (lines 179-180). It looks some participants were previously given a Diagnosis? Please clarify**

*Participants with Complex Regional Pain Syndrome were not recruited to take part in the study, they were not given a diagnosis as part of the study but if they already held a diagnosis of CRPS then they were not eligible to take part, this has been clarified in the text below:*

*“(Participants with a diagnosis of Complex Regional Pain Syndrome were not recruited to take part in the study due to research showing increasing pain after stretching illusions [30]).”* **• Line 279-281: The reviewers find a bit unclear why the first two questions are grouped under "illusory experience." Grouping the questions into Ownership (e.g., "It felt like my finger was really stretching" / "It felt like the finger I saw was part of my body") and Disownership (e.g., "It felt like the finger I saw no longer belonged to me" / "It felt like the finger I saw was no longer part of my body") might provide a clearer distinction between the positive experience of ownership and the sense of losing ownership. This would make it easier for readers to grasp the different sensory perceptions being measured.**
*We appreciate that the term “ownership” might actually make more sense than “Illusory experience”, however, previous work using the same questionnaire has grouped these statements as “Illusory Experience” and therefore to maintain consistency with previous work we need to keep the phrasing the same. Although, since it does appear to make more immediate sense to use the term “Ownership” this has been added in brackets following first mention of the Illusory experience statements to aid readers in their understanding.*

*“Two statements related to illusory experience (ownership): “It felt like my finger was really stretching” / “It felt like the finger I saw was part of my body”, two related to disownership: “It felt like the finger I saw no longer belonged to me” / “It felt like the finger I saw was no longer part of my body””* **- There is a possible error in the reference index for the study of Gilpin et al. in line 106, where the reference index is set at 24 while that same index corresponds to an uncited article by Haggard et al. in the reference list.**

*The reference list has been amended and all references cited in text should now be correct. The Haggard et al reference is now correctly cited as reference [23], which refers to the somatosensory blurring hypothesis.*  **• Data analysis: The authors performed Wilcoxon tests to compare pre- and post-pain levels across conditions, which is suitable for non-parametric data but primarily addresses group-level effects. However, given that some individuals experienced clinically meaningful pain reductions (greater than 30% or 50%), this method may overlook important individual variability in responses. A generalized linear mixed model (GLMM) would allow the authors to account for both group and individual-level variability, offering a more comprehensive analysis. By using GLMM, they could better capture the differences in pain reduction across participants, potentially revealing significant effects that the Wilcoxon test, focused on overall group trends, might not detect. This would provide a clearer picture of how the illusory conditions impacted pain on an individual basis, especially for those who experienced substantial pain relief.**

*Whilst we appreciate the suggestion of running a GLMM, and acknowledge that it might reveal significant effects regarding individual pain reduction, we do not wish to deviate from the preregistered analysis plans for analysis of pain data. The Wilcoxon tests were chosen because of their ability to assess group level differences, which is what we were expecting to find within this dataset. The individual differences were not anticipated and were why exploratory condition specific analyses were run, to see if the pain reductions were related to type of chronic pain condition. The individual nature of the pain reduction seen within this dataset would hopefully be useful information for future researchers assessing analgesia following resizing illusions, where they can then make an informed decision about potentially using a GLMM to detect individual effects.* **• While Illusory Resizing showed strong analgesic effects in some individuals, the lack of a consistent effect across all participants suggests that illusory analgesia may be present but cannot be definitively attributed to the manipulation alone. Other potential cofactors cannot be ruled out, especially given the absence of statistically significant group-level differences, contrary to the statement in lines 526-527. Please argument.**

*The discussion surrounding vibratory analgesia not being the only mechanism through which pain reduction was observed within this sample does highlight that there is likely a component of illusory resizing which might therefore underpin the analgesia effects observed, but we appreciate that this does not clearly show that illusory resizing is the only possibility for analgesia once removing the possibility of vibrations causing the pain relief. Therefore, we have added some text after the discussion regarding vibratory analgesia to highlight the possibility of cofactors influencing one’s experience of analgesia which merit further investigations:*

*"*…*since vibration was present across all conditions and more participants reported pain reductions in the illusory conditions compared to the non-illusion conditions, it is clear that there were illusory analgesic effects observed beyond that induced by vibratory analgesia. However, this does not mean that the only cause of the observed analgesia is resulting from the illusory manipulations, it is possible that there are other potential cofactors which might influence one’s likelihood of experiencing analgesia during illusory resizing, such as possible expectation / placebo effects, which merit further investigation within future research.”* **• In the conclusion (around line 552), the SSEP data showed a reduction in median amplitude in the multisensory condition, aligning with the somatosensory blurring/sharpening hypothesis. However, no significant differences were found between conditions, possibly due to some participants not experiencing illusory analgesia. If the somatosensory blurring/sharpening hypothesis is not fully supported, this raises the question: Is the theory suggesting that magnifying the hand to increase its cortical representation, and thereby reduce pain, a more fitting explanation? Or are there other possible explanations? The authors should clarify this point.**
*Clarification about no support being found for the magnification hypothesis has been added to the conclusion as can be seen below, but no note regarding other possible explanations for the SSEP effects observed here is given, since there is not a clear explanation which fits the data better than the somatosensory blurring / sharpening hypothesis.*

*“SSEP data however, despite showing a reduction in median amplitude in the multisensory condition in line with the somatosensory blurring / sharpening hypothesis, did not show overall significant differences between conditions possibly due to the lack of illusory analgesia experienced for some within the sample. This lack of group level amplitude reductions during illusory conditions, however, does not mean support was found for the magnification hypothesis, since no significant increases in median SSEP amplitudes during illusory conditions were observed either.”* **Other minor points:

• The sentence in lines 63-65 could be re-arranged or split into two sentences to increase readability.**

*This sentence has been split to increase readability and now reads:*

*“**Individuals who live with chronic pain could have a cortical misrepresentation of their body and its incoming somatosensory signals, including pain [10]. Along with this, it is possible that individuals with chronic pain might experience perceptual size dysfunctions of their affected limbs, which could underpin their persistent pain [10].”* **• The presentation of the research hypotheses in the introduction would be clearer if they were explicitly grouped into three categories: into Illusory Experience, SSEP, and Pain Reduction to simplify their comprehension.**

*A sentence has been added before mention of each hypothesis to group them into categories as can be seen below:*

*“This study had three categories of hypothesis, the first relating to illusory experience (1), the second related to SSEP responses (2a-c), and the third to pain reduction (3a-c).”* **• The statement about the importance of applying Illusory Resizing in clinical settings (lines 534-536) would have a greater impact if it were separated into its own sentence, apart from the argument about its lab use (lines 536-537).**

*This sentence has been split and reworded as can be seen below:*

*“Fig 6 shows that a substantial proportion of participants experienced either a clinically or extremally meaningful level of pain reduction following these conditions, highlighting the potential of this therapy for day-to-day treatment. Similarly, however, those experiencing an increase in pain level should not be ignored. It is, therefore, recommended that should illusory resizing be offered as a treatment for chronic hand-based pain that it is not provided within a lab setting such as the current study where significant increases in pain were found due to the experimental set up, but that home based options for delivery of resizing illusions should be prioritised.”*

**Reviewer #2:**

**1 Previous studies (e.g., Tidoni et al., 2015) found that the physical parameters of tendon vibration could be individually adjusted to induce optimal illusions. This has also been observed in clinical samples (e.g., spinal cord injury, Fusco et al., 2016). Given this, how and why did the authors select 25 Hz and 0.18 N to induce the digit resizing illusion?**

*26Hz was not chosen as a specific frequency to induce the resizing illusion, but was chosen because this was the vibration frequency found to elicit reliable steady state signals during pilot testing, which was informed by previous vibrotactile steady state research as mentioned in supporting information Fig S1. To make it clear to the reader that this was the case, additional text has been added to direct them to supporting information as can be seen below. 0.18N was simply the measured peak force for the tactor.*

*"Vibrations were delivered to the participant’s finger in all conditions using a miniature electromagnetic solenoid stimulator/bone conductor* *(Dancer Design Tactor; diameter 1.8mm) emitting vibrations produced by sending amplified 26Hz sine wave sound files, with stimulus intensity controlled by an amplifier (Dancer Design TactAmp). 26Hz was the chosen frequency after pilot testing (see Supporting Information Fig S1). The tactor was driven at 50% of the maximum (i.e., a peak input voltage of 3V) using a 26Hz sine-wave, and delivered a peak force of 0.18N.”*

**2 Since proprioceptive illusions can reflect different sensitivity or susceptibility across participants, did the authors introduce the type of illusion beforehand to establish any kind of prior expectation?**

*The participants were not exposed to the illusions prior to the experiment, which has now been made clear through added text to the experimental procedure section which can be seen below:*

*“Participants were encouraged to take a break between each of the blocks to stretch their hand. All participants were naïve to resizing illusions before taking part in the experiment.”*

**3 The authors reported that SSEPs were averaged across the activity recorded during the entire trial window. However, since the onset of the illusion may vary from trial to trial and might not correspond to time 0 (i.e., the beginning of the trial), could the null effect observed be due to varying patterns that do not consistently reflect the illusion?**

*The reviewer raises a good point here, and although it is possible that this variability contributed to the null effects observed, we did not anticipate that the SSEP would be modulated at different times for different conditions / participants, and therefore did not plan our analyses in such a way. Additionally, we would not have a sufficiently strong signal to enable this type of analysis, and without having a time-locked response from participants to indicate when the illusion occurred, it would not be possible to reanalyse in this way. We have therefore maintained out pre-registered analysis pipeline for the SSEP data but take on board this suggestion and will consider it in future SSEP work on resizing illusions where these thoughts can be had before data collection.*

**4 Related to the previous point, when did the experimenter begin pulling the finger, and how was this aligned with the individual onset of the illusion? Did participants verbally indicate when they started feeling the illusion?**

*The researcher pulled the finger when the manipulation part of the trial began, which was 1 second after the researcher initiated the start of the trail with the button box, which has now been clarified in additional text as can be seen below:*

*“**The researcher used a button press to dictate the start of the manipulation, and started pulling the finger, when needed, synchronously within the 2.4 second manipulation phase. This occurred 1 second after the researcher pressed the button to initiate the trial.”*

**5 Why did the experimenter touch the target finger without pulling it? Please clarify this aspect.**

*The non-illusion tactile (NIT) condition was included due to previous research findings that tactile input alone can reduce pain, and therefore this was included as a control to the multisensory condition. This is mentioned when the hypotheses are set out at the end of the introduction but has now also been included in the experimental procedure section as a reminder to readers, which can be seen below:*

*“Participants underwent 4 conditions: multisensory stretching (MS), unimodal-visual stretching (UV), a non-illusion control condition without tactile input (NI), and a non-illusion control condition with tactile input (NIT). There was vibrotactile stimulation to the finger in all conditions, but only tactile input of the researcher touching the participants hand in the conditions where this is mentioned. Tactile input was given in the NIT condition due to previous research suggesting that tactile input alone can reduce pain ratings [27,28].”*
